# Preliminary Safety and Efficacy of Proton Plus Carbon-Ion Radiotherapy With Concurrent Chemotherapy in Limited-Stage Small Cell Lung Cancer

**DOI:** 10.3389/fonc.2021.766822

**Published:** 2021-11-11

**Authors:** Ning-Yi Ma, Jian Chen, Xue Ming, Guo-Liang Jiang, Jiade J. Lu, Kai-Liang Wu, Jingfang Mao

**Affiliations:** ^1^ Department of Radiation Oncology, Shanghai Proton and Heavy Ion Center, Shanghai, China; ^2^ Department of Radiation Oncology, Shanghai Key Laboratory of Radiation Oncology (20dz2261000), Shanghai, China; ^3^ Department of Radiation Oncology, Shanghai Engineering Research Center of Proton and Heavy Ion Radiation Therapy, Shanghai, China; ^4^ Department of Radiation Physics, Shanghai Proton and Heavy Ion Center, Shanghai, China; ^5^ Department of Radiation Oncology, Shanghai Proton and Heavy Ion Center, Fudan University Cancer Hospital, Fudan University, Shanghai, China

**Keywords:** small cell lung cancer, radiotherapy, proton, carbon ion, efficacy

## Abstract

**Objectives:**

This study aimed to investigate the tolerance and effect of proton plus carbon-ion radiotherapy with concurrent chemotherapy in limited-stage small cell lung cancer using the pencil beam scanning technique.

**Materials and Methods:**

From March 2017 to April 2020, 25 patients with limited-stage small cell lung cancer treated with combined proton and carbon-ion radiotherapy were analyzed. The primary lesions and involved lymph nodes were irradiated using 2–4 portals. Proton and sequential carbon-ion beams were delivered with a median dose of 67.1 (range, 63–74.8) GyE as fraction doses of 2.0–2.2 GyE with proton beams in 20–23 fractions and 3.0–3.8 GyE with carbon ions in 5–8 fractions. Chemotherapy was delivered concurrently with radiotherapy in all patients.

**Results:**

At the last follow-up, the 2-year overall and locoregional progression-free survival rates were 81.7% and 66.7%, respectively. Radiochemotherapy was well tolerated, with grade 1, 2, and 3 acute toxicities occurring in 12.0%, 68.0%, and 20.0% of patients, respectively. All grade 3 acute toxicities were hematologically related changes. One patient experienced grade 3 acute non-hematological toxicity in the esophagus, and one other patient had grade 3 bronchial obstruction accompanied by obstructive atelectasis as a late side effect.

**Conclusion:**

Proton plus carbon-ion radiotherapy using pencil beam scanning yielded promising survival rates and tolerability in patients with limited-stage small cell lung cancer. A prospective clinical study is warranted to validate the therapeutic efficacy of particle radiotherapy in combination with chemotherapy in limited-stage small cell lung cancer.

## Introduction

Lung cancer is the second most common malignant tumor and leading cause of cancer deaths worldwide (1, 2). Small cell lung cancer (SCLC) accounts for 13%–17% of all lung cancer cases (3). As SCLC is a poorly differentiated neuroendocrine carcinoma with poor prognosis, its biological behavior is generally far more aggressive than non-SCLC (4, 5). Most patients are diagnosed in the late stage, and limited-stage SCLC (LS-SCLC) only accounts for approximately one-third of all patients at the first diagnosis (3).

SCLC is sensitive to chemotherapy, which has been the cornerstone of treatment for this disease for the last 40 years (6–8). Furthermore, based on the results of various meta-analyses, the addition of radiotherapy (RT) can improve overall survival (OS) and local disease control in patients with LS-SCLC, resulting in a 25%–30% decrease in the local failure rate and a 5%–7% increase in the 2-year OS rate (9, 10). Currently, concurrent chemoradiotherapy is the standard initial treatment for LS-SCLC (11), and RT administered concurrently with the first/second cycle of chemotherapy is highly recommended (12, 13). Hyperfractionated RT (HFRT) of 45 Gy delivered at 1.5 Gy twice daily or conventional RT of 60–70 Gy at 2.0 Gy once daily has shown comparable survival and been recommended for LS-SCLC (14–17). Although SCLC is both radiosensitive and chemosensitive, nearly 30% of the patients with LS-SCLC experience local failure, even after standard chemotherapy and HFRT (14). Recently, a phase 2 study demonstrated that thoracic HFRT of 60 Gy at 1.5 Gy twice daily leads to an improved median OS duration and 2-year OS rate, as well as similar adverse effects, when compared with a dose of 45 Gy in LS-SCLC (18). Seemingly, increasing the radiation dose can lead to an improvement in the clinical outcome if the toxicity is tolerable. Therefore, a technical improvement in a highly conformal therapy may provide a higher RT dose to the malignant lesions while avoiding the severe side effects of high-dose administration to the surrounding organs at risk (OARs).

Particle RT, that is, proton and carbon-ion beam RT, has been developed globally in the last 30 years. It has the physical advantage of Bragg peaks and is, thus, a technical improvement over the highly conformal therapy of photon (19). Proton RT (PRT) in LS-SCLC has yielded promising local control (LC) and OS, along with tolerable side effects, compared with photon RT combined with chemotherapy (20, 21). With the special radiobiological properties of high linear energy transfer irradiation, carbon-ion beams are much more effective than X-rays or PRT in terms of DNA damage and exhibit excellent outcomes in patients with hepatocellular carcinoma, bone or soft-tissue sarcomas, prostate cancer, head and neck cancers, and non-SCLC (19, 22, 23). Theoretically, PRT has similar radiobiological effectiveness as X-ray RT and can eliminate most radiosensitive tumor cells while sparing a greater proportion of the healthy tissues surrounding the tumor as compared with X-rays. The remaining radioresistant subgroup of cancer cells or cancer stem cells, whose enrichment may explain the treatment resistance, aggressiveness, recurrence, and metastasis of SCLC (24), can be further eliminated by subsequent carbon-ion RT (CIRT), which offers additional potential radiobiological advantages. However, it has not been confirmed by clinical practice.

Therefore, it is worth investigating whether using combined PRT and CIRT concurrently with chemotherapy could improve the outcome in patients with LS-SCLC. Our center is one of the few that can produce both proton and carbon-ion beams with a synchrotron. Promising clinical outcomes in bone or soft-tissue sarcomas, head and neck cancers, and early-stage non-SCLC have been obtained over the last 5 years (25–28). In this study, we retrospectively investigated the tolerance and effect of PRT and sequential CIRT using the pencil beam scanning technique in combination with chemotherapy in LS-SCLC.

## Materials and Methods

### Patient Selection Criteria

Patients who satisfied the following selection criteria were enrolled in this retrospective analysis: histologically confirmed SCLC; limited stage evaluated by contrast-enhanced chest computed tomography (CT), brain magnetic resonance imaging (MRI), and positron emission tomography (PET)/CT before RT; received PRT and sequential carbon-ion boost RT concurrently with chemotherapy. Patients were excluded if distant failure had occurred after initial chemotherapy or if they had previously undergone surgery or thoracic irradiation. This study was carried out in accordance with the Declaration of Helsinki and approved by our institutional review board, and written informed consent was obtained from all the participants.

### Treatment Regimen

Chemotherapy, comprising a total of 4–6 cycles of etoposide and platinum, was administered. The administration of concurrent thoracic PRT and CIRT before the third cycle of chemotherapy has been highly recommended for LS-SCLC. Prophylactic cranial irradiation (PCI) was given after the completion of chemoradiotherapy.

### Thoracic Proton Radiotherapy and Carbon-Ion Radiotherapy

The movement of the solid tumor was tracked in all patients using fluoroscopy or four-dimensional (4D) CT before simulation CT scan. If the lesion moved more than 5 mm in the whole respiratory cycle, only the phase in which the tumor motion was, within 5 mm, would be available for active delivery, which was achieved by adapting the active breathing control (ABC) or respiratory gating technique. Other patients were irradiated under a free-breathing condition. Identical respiratory control was applied in either particle treatment or CT simulation to irradiate the involved lesions; only the involved lesions were irradiated. The gross tumor volume (GTV) of the involved lesions was detected on thoracic CT and PET/CT. During the implementation of the respiratory control device, the GTVs were segmented on the reconstructed CT images in the 10 respiratory phases during 4D CT simulation. The involvement of these GTVs in the 10 phases was defined as the internal GTV (iGTV). The clinical target volume (CTV) was the expansion on the GTV/iGTV with a 0.6–0.8-cm margin. An additional 0.5–0.7-cm margin on the lateral side and a 0.7–1.5-cm margin along the beam entry direction were implemented on the CTVs for its planning target volume (PTV); the beam-specific margin-expanding rules account for the range and position to offset uncertainties. The definitions of all target volumes were the same for both PRT and CIRT. The treatment plans were designed in the Syngo treatment planning system (Siemens Health Care Systems, Erlangen, Germany). Doses were prescribed as GyE (gray equivalent to photon) based on the Local Effect Model embedded in the Syngo system. During planning optimization, according to the priority of the delivery robustness or the dose constraints to the critical organs, single-beam optimization or intensity-modulated particle therapy techniques were applied. There was a demand in the dose coverages to the target, wherein at least 99% of the GTV/iGTV was covered by 95% of the prescription dose, 99% of the CTV was covered by 95% of the prescription dose, and 90% of the PTV was covered by 90% of the prescription dose during treatment planning. Only when the malignant tumors were adjacent to the OARs did the expanded margins become compromised in terms of treatment safety. The RT was delivered based on 2D image-guided verification 5 days per week. The simulation CT scans were reviewed weekly for each patient during the RT to recalculate anatomic changes and dose distribution. Replanning was required when inadequate coverage of the tumor targets or overdosing to the OARs was detected after recalculation.

### Follow-Up and Statistical Analyses

Patient follow-up involved weekly physical examinations, complete blood counts, and monthly hepatic/renal function tests during irradiation. Follow-ups were scheduled every 3–4 months after irradiation during the first 2 years, every 6 months during years 3 and 4, and annually thereafter.

The endpoints included the following: treatment-related toxicities, evaluated by the Common Terminology Criteria for Adverse Events version 4.0; tumor responses, evaluated by imaging (CT, MRI, and PET), or pathological examination with endoscopy or aspiration biopsy if disease relapse was suspected; and the median times of OS, progression-free survival (PFS), locoregional PFS (LPFS), and distant metastasis-free survival (DMFS) (1- and 2-year rates). The observation for all endpoints began at the initiation of RT until an event of interest occurred or until the last follow-up.

The rates of OS, PFS, LPFS, and DMFS were estimated using the Kaplan–Meier method. All statistical analyses were performed using the STATA statistical software package version 11.0 (StataCorp LP, College Station, TX, USA).

## Results

### Patient and Treatment Characteristics

From March 2017 to April 2020, 25 consecutive patients who met the selection criteria were included in this study. The patients’ clinical and treatment characteristics are listed in **Table 1**.

**Table 1:** Characteristics of the patients with limited-stage small cell lung cancer included in this study.

Age (years), median [range]	60.8 [30.8–77.6]
Sex, no. (%)	
Male	22 (88.0)
Female	3 (12.0)
Smoker, no. (%)	
Current/former ≥20 pack-years	17 (68.0)
Current/former <20 pack-years	1 (4.0)
No	7 (28.0)
Location of lesion, no. (%)	
Right upper lobe	7 (28.0)
Right middle lobe	2 (8.0)
Right lower lobe	6 (24.0)
Left upper lobe	6 (24.0)
Left lower lobe	4 (16.0)
AJCC stage, no. (%)	
II	4 (16.0)
III	21 (84.0)
Karnofsky performance status at diagnosis, median [range]	90 [80–100]
Induction chemotherapy	
No. (%)	25 (100.0)
No. of cycles, median [range]	2 [1–5]
Concurrent chemotherapy	
No. (%)	25 (100.0)
No. of cycles, median [range]	1 [1–2]

AJCC, American Joint Committee on Cancer.

Chemotherapy was administered to all 25 patients, with a median of 6 (range, 4–8) cycles. Pre-RT chemotherapy was delivered to all patients: etoposide and platinum chemotherapy to 21 patients; etoposide monotherapy to one; etoposide and platinum, followed by irinotecan plus cisplatin to one; and etoposide and platinum plus pembrolizumab or bevacizumab to two. Thoracic particle RT was delivered after a median of 2 (range, 1–5) cycles of pre-RT chemotherapy; 11 patients initiated their RT after three cycles. The median interval from the initiation of anticancer therapy until the end of RT (SER) was 13.3 (range, 9.1–22.0) weeks. Concurrent chemotherapy was delivered with a median of 1 (range, 1–2) cycle, including etoposide and cisplatin in 16 patients, etoposide and carboplatin in six, irinotecan and cisplatin in one, and etoposide and nedaplatin in two.

Thoracic PRT and CIRT were delivered sequentially using the pencil beam technique *via* a synchrotron facility. A breathing-control technique was used in all patients—either ABC (one patient) or respiratory gating (24 patients). Particle beams were administered with a median dose of 67.1 (range, 63–74.8) GyE over a median of 29 (range, 25–30) fractions. An RT regimen of 65–67.1 GyE/29–30 fractions was used in 15 patients whose total dose was similar to that of a PRT prospective study. Additionally, one patient received a higher-dose RT of 74.8 GyE/30 fractions after 1 week of treatment interruption due to equipment failure. In the remaining nine patients, a regimen of 63 GyE/25 fractions was used to shorten the treatment interval. Proton beam was administered in a median dose of 44 GyE (44–48.4) in 22 fractions (20–23), with 2 GyE or 2.2 GyE per fraction. Carbon-ion beam was administered in a median dose of 23.1 (range, 19–26.4) GyE in 7 fractions (5–8), including 21–23.1 GyE/7 fractions in 15 patients, 26.4 GyE/8 GyE fractions in one, and 19 GyE/5 fractions in nine. Plan recalculation based on the weekly simulation CT was regularly undertaken in each patient to verify the dose distribution to the tumor target and OARs. PRT and CIRT replanning was performed in five patients when inadequate coverage to the tumor targets or overdosing to the OARs was detected. PCI combined with thoracic PRT and CIRT was administered to 21 patients.

**Figure 1:**
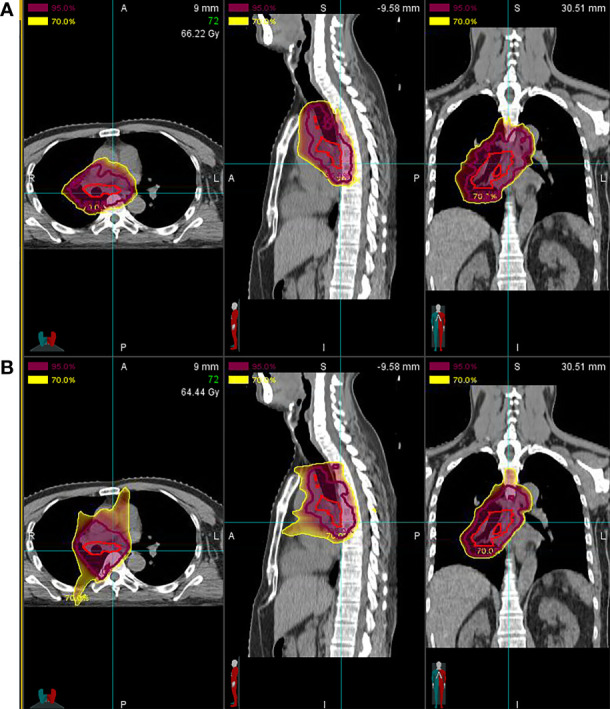
Comparison of treatment planning for particle *vs*. X-ray radiotherapy (RT). **(A)** Proton radiotherapy (PRT) and carbon-ion radiotherapy (CIRT) show a significant decrease in the average doses to the heart and lungs compared with **(B)** X-ray RT [mean heart dose, 13.95 gray equivalent to photon (GyE) *vs*. 16.40 Gy; mean lung dose, 9.12 GyE *vs*. 21.46 Gy].

The dosimetric parameters of OARs in all cases are summarized in **Table 2**. Dose distribution to the OARs was relatively low even in centrally located primary lesions and the involvement of mediastinal lymph nodes. In particular, the mean values of the doses to the lungs, ipsilateral lung, and contralateral lung were 11.62 (range, 7.98–18.67), 19.55 (range, 13.51–26.99), and 2.61 (range, 0.04–12.20) GyE, respectively, and the percentage of volume (both lungs) receiving >5 or 20 Gy was 36.18% (range, 20.13%–55.26%) and 23.01% (range, 14.58%–41.54%), respectively. The treatment planning of particle RT showed an improvement in the average dose to the heart and lungs compared with X-ray RT (**Figure 1**).

**Table 2:** Dosimetric parameters of thoracic radiotherapy for the organs at risk.

Organ at risk	Dosimetric parameter	Mean ± SD
Lungs	Dmean (GyE)	11.62 ± 2.56
	V5 (%)	36.18 ± 9.86
	V20 (%)	23.01 ± 5.92
Ipsilateral lung	Dmean (GyE)	19.55 ± 3.09
Contralateral lung	Dmean (GyE)	2.61 ± 3.09
Heart	Dmean (GyE)	5.49 ± 2.52
Esophagus	Dmax (GyE)	63.42 ± 17.20
Spinal cord	Dmax (GyE)	20.06 ± 11.16

SD, standard deviation; Gy, gray; GyE, gray equivalent to photon; Dmean, mean dose; Dmax, maximum dose; V5, percentage of volume receiving >5 Gy; V20, percentage of volume receiving >20 Gy.

### Treatment-Related Toxicities

In our study, the treatment was well-tolerated by patients. PRT and sequential CIRT were completed in all patients. RT was suspended for ≥3 days owing to hematological toxicities (one patient), esophagitis (one patient), treatment replanning (one patient), or unplanned failure of RT facility (two patients).

Grade 1, 2, and 3 acute toxicities occurred in 12.0%, 68.0%, and 20.0% of all cases, respectively (**Table 3**). There were no grade ≥3 non-hematological adverse effects, except in a patient who experienced grade 3 acute radiation esophagitis during PRT. Additionally, another patient experienced grade 3 bronchial obstruction accompanied by obstructive atelectasis as a late adverse effect. This patient had received one additional fraction of carbon-ion irradiation, and the total dose reached 74.8 GyE in 30 fractions because RT had been suspended for a week due to an unforeseen failure of the RT facility. Grade 1 late dermatitis and esophagitis were recorded in two patients each. Radiation-induced late lung injuries of grades 1 and 2 were recorded in 15 (60.0%) and six (24.0%) patients, respectively.

**Table 3:** Frequencies of treatment-related acute adverse events according to the Common Terminology Criteria for Adverse Events version 4.0.

	Grade, no. (%)				
	1	2	3	4	5
Pulmonary					
Cough	6 (24.0)	0	0	0	0
Pneumonitis	2 (8.0)	1 (4.0)	0	0	0
Gastrointestinal					
Esophagitis	15 (60.0)	1 (4.0)	1 (4.0)	0	0
Cardiac					
Tachycardia	4 (16.0)	0	0	0	0
General					
Fever	2 (8.0)	0	0	0	0
Weight loss	2 (8.0)	0	0	0	0
Dermatitis	6 (24.0)	0	0	0	0
Hematological					
Leukopenia	4 (16.0)	15 (60.0)	1 (4.0)	0	0
Neutropenia	2 (8.0)	11 (44.0)	5 (20.0)	0	0
Anemia	13 (52.0)	8 (32.0)	0	0	0
Thrombocytopenia	4 (16.0)	5 (20.0)	0	0	0

### Clinical Outcomes

**Figure 2:**
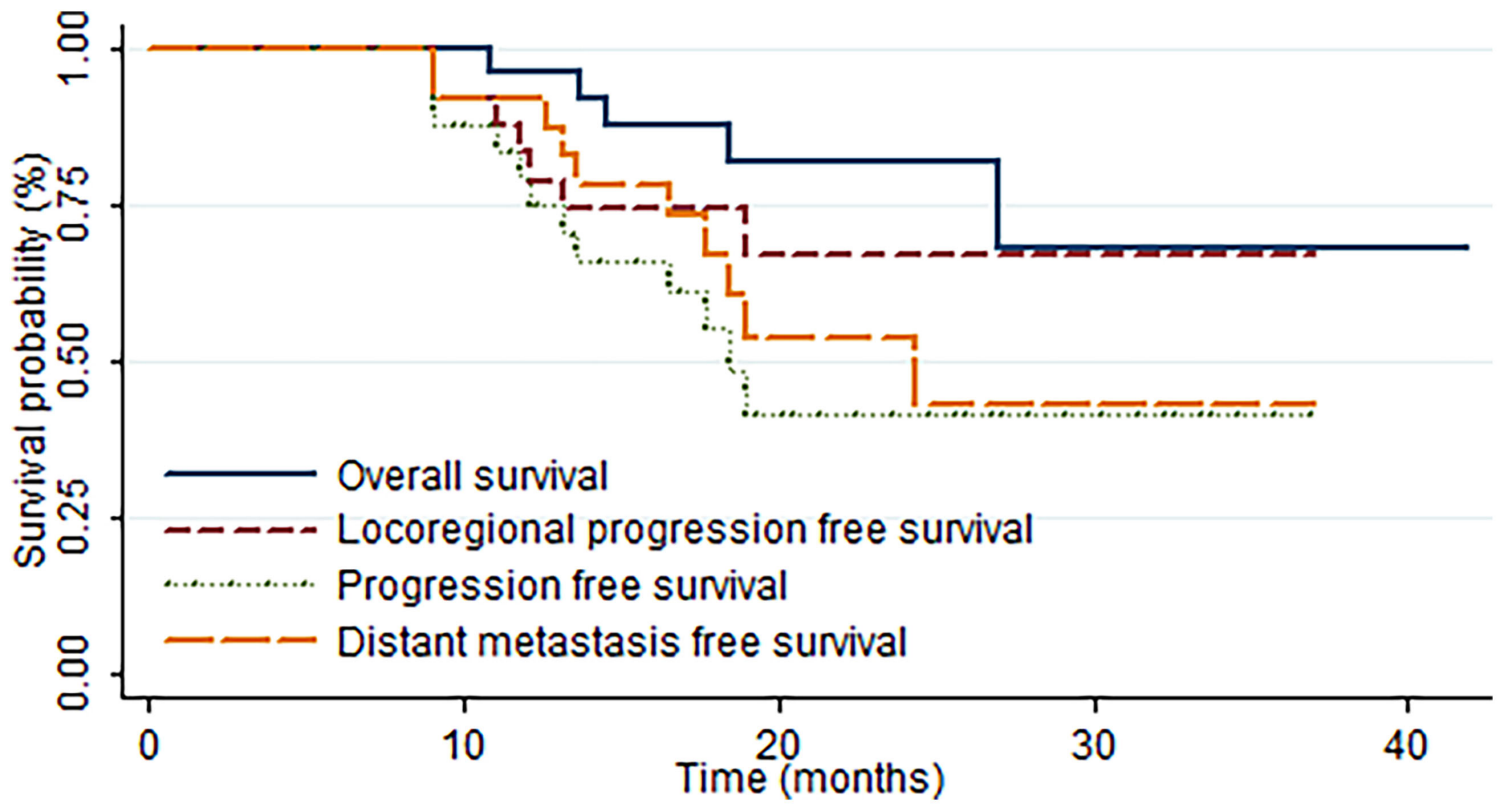
Kaplan–Meier estimates of the rates of overall survival (OS), progression-free survival (PFS), locoregional progression-free survival (LPFS), and distant metastasis-free survival (DMFS) in all patients.

The median OS and LPFS times were not achieved at the last follow-up. Meanwhile, the median DMFS and PFS times were 24.4 and 18.4 months, respectively. With a median follow-up time of 19.2 (range, 6.7–41.9) months, the 1-year OS, LPFS, DMFS, and PFS rates were 95.8%, 83.1%, 91.7%, and 79.0%, respectively, and the 2-year OS, LPFS, DMFS, and PFS rates were 81.7%, 66.7%, 53.6%, and 41.2%, respectively (**Figure 2**).

Moreover, treatment failure was noted in 12 patients at the last follow-up. Two (8.0%), five (20.0%), and five (20.0%) patients had locoregional progression, distant metastasis, and both, respectively. The metastatic sites were the brain in six patients, the lung in three patients, and the liver and bone in three.

## Discussion

Nowadays, concurrent chemotherapy and RT are the standard of care for LS-SCLC. There is no consensus reached in terms of the optimal and exact timing of chemotherapy and RT or dose and fractionation of RT. Early thoracic photon RT was recommended in patients with LS-SCLC, with HFRT of 45 Gy with 1.5 Gy twice daily or conventional RT of 60–70 Gy with 2.0 Gy once daily (14–17). It was suggested that RT administered concurrently with the first/second cycle of chemotherapy (29) and shorter duration of SER (<30 days) were associated with improved survival (13). Two studies have reported the efficacy of PRT in treating LS-SCLC. The prospective clinical trial performed by Rwigema et al. (20) included 30 patients with LS-SCLC who were administered 45 GyE/30 fractions of proton HFRT or 59.4–66.6 Gy/33–37 fractions of conventional RT, along with concurrent chemotherapy of cisplatin or carboplatin plus etoposide. The results revealed that the median OS and PFS times were 28.2 and 14.3 months, respectively, and the 1- and 2-year OS, PFS, and LC rates were 71.5%, 63.0%, and 85% and 57.6%, 42.0%, and 68.6%, respectively. Moreover, the PRT-induced non-hematological toxicity rates were lower, with 13.3% of grade ≥3 toxicities, than those with photon RT (14, 17). Colaco et al. (21) retrospectively analyzed PRT in six patients with LS-SCLC amounting to 60–66 cobalt gray equivalent (CGE)/30–34 fractions of conventional RT or 45 CGE/30 fractions of HFRT. The 1-year PFS and OS rates were 66% and 83%, respectively, without grade ≥3 non-hematological toxicities.

Nevertheless, obtaining long-term LC and prolonging survival using current proton or photon chemoradiotherapy in LS-SCLC remain a challenge. The enrichment of cancer stem cells may contribute to treatment resistance, aggressiveness, recurrence, and SCLC metastasis (24). Theoretically, sequential carbon-ion beams can increase the possibility of eliminating the remaining radioresistant subgroup of cancer cells or cancer stem cells after X-ray or PRT. To the best of our knowledge, this study is the first assessment of PRT plus sequential CIRT in LS-SCLC in clinical practice.

A dosimetric comparison study with intensity-modulated RT (IMRT) showed that a PRT or CIRT plan significantly reduced the doses to the lungs, esophagus, and other thoracic organs, as well as the volumes of the received 5–60 Gy in lung cancer (30–32). Colaco et al. (21) analyzed PRT and IMRT in six patients and found that proton therapy spared the lung and esophagus better than IMRT did. The median differences of the mean dose / V5 to the lungs and esophagus between IMRT and PRT were 5 Gy / 17% and 2.6 Gy / 10%, respectively. Rwigema et al. (20) analyzed PRT and IMRT in 30 patients, and results showed that proton therapy demonstrated a significant improvement in V5 and the mean dose to the lungs, heart, and spinal cord. Our study on the combination of proton and carbon ion showed comparable or favorable dosimetry of the mean doses to the lungs and heart, V5 and V20 for the lungs, and maximum dose to the spinal cord to the abovementioned studies. The dosimetric improvement of particle RT to normal tissue/organs may explain the minority of severe acute radiation-induced toxicities (33). A mean dose of <10 GyE to the heart in all the patients in our study also contributed to the decrease in related clinical toxicities, as mean doses of ≥10 Gy to the heart were associated with a significantly higher percentage in the rate of major coronary events than those of <10 Gy, as reported in patients with breast cancer on long-term follow-up after thoracic RT (34).

Our study also showed that the treatment-related toxicities of particle therapy with concurrent chemotherapy were mild and well-tolerated by patients. No grade 4–5 toxicities were observed. Acute hematological toxicities of grade 3 were observed in 20% of the cases of the entire group. Despite the inclusion of mediastinal and/or pulmonary hilar regions, no grade ≥3 pulmonary acute side effects occurred. This may be due to the relatively low doses delivered to the OARs (mean dose to both lungs <20 GyE was achieved in all patients, and the median value was only 11.1 GyE). Only one patient experienced a grade 3 non-hematological acute event in the esophagus. Another patient experienced grade 3 bronchial obstruction accompanied by obstructive atelectasis as a late side effect. This patient received one additional fraction of CIRT, to a total dose of 74.8 GyE in 30 fractions, after 1 week of treatment interruption due to equipment failure—this was the highest maximum dose administered to the bronchus in the entire cohort. The adverse effects in our study were milder than those in the prospective PRT study on lung cancer (20). The latter reported incidences of 3.3%, 3.3%, 3.3%, and 3.3% of grade ≥3 radiation-induced pneumonia, esophagitis, anorexia, and pericardial effusion, respectively, and 43.3%, 43.3%, 23.3%, and 10% of grade ≥3 lymphopenia, neutropenia, anemia, and thrombocytopenia, respectively, after HFRT (45 GyE) or conventional RT (59.4–66.6 GyE) (20). Modern photon-based RT for LS-SCLC induced grade ≥3 pneumonitis and esophagitis as high as 5%–10% and 30%, respectively (12, 14, 35–37). Furthermore, the use of pencil beam scanning led to a much lower possibility of dermatitis in our study compared with the use of passive beam scattering (grade 1, 24% *vs*. 38%) (38).

Although RT was delivered after three cycles of chemotherapy in two-fifths of the patients and the SER reached 3.1 (range, 2.1–5.1) months, our results revealed promising OS rates with lower toxicities when using proton and carbon-ion beams as the resources of RT during concurrent chemoradiotherapy, which was numerically favorable when compared with the findings in prior publications on photon/proton-based chemoradiotherapy (**Table 4**). Considering that PRT and CIRT combined with chemotherapy may improve the outcomes with acceptable toxicities in LS-SCLC, a combination of PRT, CIRT, and chemotherapy could be one of the treatment options for these patients with LS-SCLC.

**Table 4:** Efficiency and safety of photon or particle radiotherapy for limited-stage small cell lung cancer.

Resource	Number	Radiotherapy	Follow-up (months)	Dose	Median OS	Median PFS	Survival rate				Toxicity (grade ≥3 non-hematological)
								1 year	2 years	5 years	
Komaki et al., 2012^a^ (39)	71	Photon	19.0	61.2 Gy/34 fractions	19	9.9	OS	−	36.6	−	Acute
							PFS	−	19.7	−	Esophageal toxicity 18.3
											Pulmonary toxicity 12.7
											Late
											Esophageal toxicity 1.4
											Pulmonary toxicity 11.3
Bogart et al., 2004^b^ (17)	63	Photon	24.7	70 Gy/35 fractions	22.4	13.4	OS	−	48	−	Esophageal toxicity 21
							PFS	−	31	−	Pulmonary toxicity 5
Takada et al., 2002^c^ (12)	231	Photon	−	45 Gy/30 fractions	19.7/27.2	−	OS	−	35.1/54.4	18.3/23.7	Esophagitis 4/9
Murray et al., 1993^d^ (40)	308	Photon	60	40 Gy/15 fractions	21.2/16	15.4/11.8	OS	−	40/33.7	20/11	Esophagitis 14.8/7.6
							PFS	−	−	(3 years) 26/19
Sun et al., 2013^e^ (41)	219	Photon	59.4	52.5 Gy/25 fractions	24.1/26.8	12.4/11.2	OS	−	50.7/56.0	24.3/24.0	Esophagitis 3.6/0.9
							PFS	51.8/48.1	28.0/23.5	−	Pneumonitis 4.5/2.8
Jeremic et al., 1997^f^ (35)	103	Photon	−	54 Gy/36 fractions	34/26	–	OS LPFS	90/71 94/74	71/53 90/69	30/15 58/37	Acute Esophageal toxicity 28.8/25.5 Pulmonary toxicity 1.9/0 Late Esophageal toxicity 1.9/2.0 Pulmonary toxicity 1.9/0
Work et al., 1997^g^ (42)	199	Photon	–	40 - 45 Gy/22 fractions	10.5/12.0	–	OS LC^h^	- -	20/19 28/32	11/12 23/27	–
Turrisi et al., 1999^i^ (36)	417	Photon	96	45 Gy/30 fractions	23/19	−	OS	−	47/41	26/16	Esophagitis 32/16
				45 Gy/25 fractions			PFS	−	29/24	−	Pulmonary toxicity 6/4
Faivre-Finn et al., 2017^i^ (14)	547	Photon	45	45 Gy/30 fractions	30.0/25.0	15.4/14.3	OS	−	56.0/51.0	34.0/31.0	Acute
				66 Gy/33 fractions			LPFS^j^	−	45.8/41.5	−	Esophagitis 19/19 Pneumonitis 3/2 Late Esophagitis 0/2 Pneumonitis 2/3
Grønberg et al., 2021^k^ (18)	170	Photon	49	60 Gy/40 fractions	37.2/22.6	18.6/10.9	OS	−	74.2/48.1	−	Esophagitis 21/18
				45 Gy/30 fractions			PFS ^j^	−	42.7/32.1	−	Pneumonitis 3/0
Colaco et al., 2013 (21)	6	Proton	12.0	45 CGE/30 fractions	−	−	OS	83			Esophagitis 0
				60–66 CGE/30–34 fractions			PFS	66			Pneumonitis 0
			
Rwigema et al., 2017 (20)	30	Proton	14.0	45 CGE/30 fractions	28.2	14.3	OS	71.5	57.6		Esophagitis 3.3
				59.4–66.6 CGE/33–37 fractions			PFS	63.0	42.0		Pneumonitis 3.3
							LC	85.0	68.6	

a Photon, 61.2 Gy (daily, 1.8-Gy fractions on days 1–22, then twice daily, 1.8-Gy fractions on days 23–33).

b Photon, 70 Gy (daily, 2-Gy fractions).

c Photon, sequential or concurrent radiotherapy.

d Photon, early (weeks 3–6 after the start of chemotherapy) vs. late radiotherapy (weeks 15–18).

e Photon, early (during first chemotherapy) radiotherapy or late (during third chemotherapy) radiotherapy.

f Photon, early (weeks 1–4 concurrent with chemotherapy) vs. late radiotherapy (weeks 6–9).

g Photon, early (before sequential chemotherapy) vs. late (weeks 18 after the start of sequential chemotherapy) radiotherapy.

h LC, % without in-field chest recurrence.

i Photon, twice daily or once daily.

j Estimated.

k Photon, high dose (twice daily) vs. standard dose (twice daily).

CGE, cobalt gray equivalent; LPFS, local progression-free survival; OS, overall survival; LC, local control; PFS, progression-free survival.

This study is the first to investigate PRT plus CIRT in LS-SCLC; however, limitations were obvious, especially the retrospective nature of the study and small sample of patient population. Prospective studies with more patients were warranted.

In summary, PRT and CIRT by pencil beam scanning yielded promising survival and tolerability in patients with LS-SCLC. Based on the preliminary results, a prospective clinical trial was undertaken to validate the therapeutic efficacy of particle RT in LS-SCLC.

## Data Availability Statement

The raw data supporting the conclusions of this article will be made available by the authors without undue reservation.

## Ethics Statement

The studies involving human participants were reviewed and approved by the Shanghai Proton and Heavy Ion Center Review Board. The patients/participants provided their written informed consent to participate in this study.

## Author Contributions

JM participated in the study design, data interpretation, reviewing and editing of the article, and funding acquisition. N-YM participated in the data analysis, writing of the article, and funding acquisition. JC participated in the data collection, analysis, and writing of the article. XM participated in the data analysis and treatment planning data collection. K-LW participated in the study design and reviewing and editing of the article. JL participated in the study design and reviewing. G-LJ participated in the study design and reviewing and editing of the article. All authors contributed to the article and approved the submitted version.

## Funding

The study was supported by grant funds from the National Key Research and Development Program of China (project no. 2018YFC0115700), Shanghai Pudong New Area Science and Technology Development Fund for the People’s Livelihood Research Project (grant number PKJ2018-Y48), Shanghai Shen Kang Hospital Development Center Clinical Science and Technology Innovation Project (grant number SHDC12019X25), Shanghai Shen Kang Hospital Development Center New Frontier Technology Joint Project of Municipal Hospital (grant number SHDC12017114), Shanghai Municipal Health Commission Funds (grant number 201940334), and Shanghai Science and Technology Development Funds (grant number 20ZR1453300). The sponsors have no involvement in the study design, analysis and interpretation of data, or writing of the article for publication.

## Conflict of Interest

The authors declare that the research was conducted in the absence of any commercial or financial relationships that could be construed as a potential conflict of interest.

## Publisher’s Note

All claims expressed in this article are solely those of the authors and do not necessarily represent those of their affiliated organizations, or those of the publisher, the editors and the reviewers. Any product that may be evaluated in this article, or claim that may be made by its manufacturer, is not guaranteed or endorsed by the publisher.
